# The end protection problem—an unexpected twist in the tail

**DOI:** 10.1101/gad.344044.120

**Published:** 2021-01-01

**Authors:** Phil Ruis, Simon J. Boulton

**Affiliations:** The Francis Crick Institute, London NW1 1AT, United Kingdom

**Keywords:** DNA damage response, NHEJ, TRF2, pluripotency, somatic cells, t-loops, telomeres

## Abstract

In this perspective, we introduce shelterin and the mechanisms of ATM activation and NHEJ at telomeres, before discussing the following questions: How are t-loops proposed to protect chromosome ends and what is the evidence for this model? Can other models explain how TRF2 mediates end protection? Could t-loops be pathological structures? How is end protection achieved in pluripotent cells? What do the insights into telomere end protection in pluripotent cells mean for the t-loop model of end protection? Why might different cell states have evolved different mechanisms of end protection? Finally, we offer support for an updated t-loop model of end protection, suggesting that the data is supportive of a critical role for t-loops in protecting chromosome ends from NHEJ and ATM activation, but that other mechanisms are involved. Finally, we propose that t-loops are likely dynamic, rather than static, structures.

## Abstract (precis)

In this review, Ruis and Boulton provide an updated t-loop model of chromosome end protection, suggesting that the data is supportive of a critical role for t-loops in protecting chromosome ends from NHEJ and ATM activation, but that other mechanisms are involved.

Chromosome ends present a dual danger to cells. Semi-conservative DNA replication is unable to replicate the extreme chromosome terminus and, because chromosome ends resemble DNA breaks, these ends can potentially activate the DNA damage response (DDR) and elicit misrepair. Mammalian chromosome ends mitigate these dual dangers through telomeres, nucleoprotein structures consisting of double-stranded (ds) TTAGGG repeats culminating in a single-stranded (ss) G-rich 3′ overhang that are bound by the hexameric shelterin complex (Fig. 1A). Shelterin facilitates the addition of new telomeric repeats by regulating the reverse transcriptase telomerase and also inhibits at least seven distinct mechanisms of DNA repair, counteracting telomere loss and DDR activation at chromosome ends, respectively.

**Figure 1.**
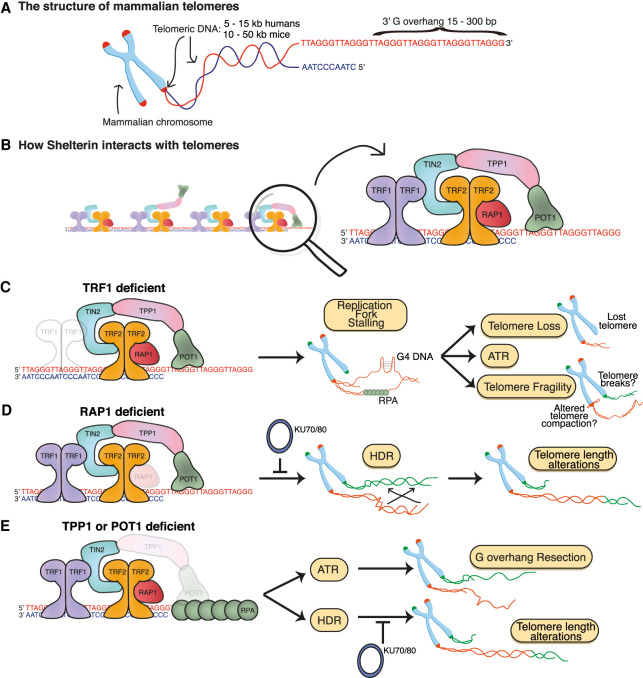
The mammalian telomere. (*A*) Depiction of a mammalian chromosome highlighting key features of the telomeres that cap each chromosome end. (*B*) Depiction of how the hexameric complex shelterin associates with telomeres. (*C*) TRF1 promotes efficient telomere replication, avoiding replication fork stalling that leads to ATR activation, telomere loss and telomere fragility. (*D*) RAP1 represses homologous recombination (HDR) within mammalian telomeres, avoiding deleterious telomere length alterations. (*E*) TPP1 recruits POT1 to telomeres, with POT1 repressing ATR activation and HDR, restricting excessive G overhang resection and telomere length alterations respectively.

Understanding how telomeres protect the ends of chromosomes from DNA damage signaling has been a focus of the telomere field for >20 yr. Since the seminal discovery that removal of the shelterin component TRF2 from telomeres leads to telomere end-to-end fusions, senescence and cell death, the question of how shelterin—and, especially, TRF2—protects chromosome ends from DNA repair activities has been paramount. A succession of papers followed, showing that loss of TRF2 leads to activation of the DNA double strand break (DSB) kinase ATM and classical nonhomologous end joining (NHEJ) at telomeres. The question of how TRF2 protects chromosome ends has thus focused on how TRF2 prevents the activation of these dual pathways—ATM and NHEJ—at telomeres. Both pathways rely on the binding of DSB sensors (MRE11/RAD50/NBS1 [MRN] and KU70/80, respectively) to the same substrate (DNA ends), suggesting that one possible way TRF2 could inhibit these pathways would be to hide these DSB ends.

The field has now broadly coalesced around the t-loop model of end protection, which hypothesises that TRF2 mediates the invasion of the single stranded telomere terminus into the chromosome-proximal telomeric dsDNA, or stabilizes this structure, sequestering the chromosome end in a lariat loop structure, elegantly hiding it from the DSB end sensors that trigger ATM and NHEJ activation. This model was first proposed in 1999 and, to a large extent, fits with published data. In particular, telomere loops have been observed via both electron microscopy (EM) and, more recently, super-resolution microscopy. Moreover, TRF2 has been shown to stimulate the formation of these t-loops in vitro, while the depletion of TRF2 leads to the reduction of these loops in cells. Although this makes TRF2-mediated t-loop formation a plausible candidate for the mechanism through which TRF2 protects chromosome ends, it has remained impossible to test this directly. Indeed, some have suggested that these t-loops are a pathological structure formed through aberrant telomere recombination. Furthermore, TRF2 interacts directly with multiple ATM and NHEJ components, and TRF2 has also been suggested to mediate telomere compaction, providing alternative means through which it could mediate end protection. Therefore, the exact role of t-loops and TRF2 in end protection has remained controversial. One key test, identifying whether t-loops protect chromosome ends in the absence of TRF2, has never before been possible as no factor, other than TRF2, has been found that can mediate t-loop formation and/or stabilization.

Several unexpected new discoveries have enabled progress to be made in this direction. Surprisingly, together with the Cesare laboratory (Ruis et al. 2020), we recently identified that in mouse embryonic stem cells (ESCs), chromosome end protection is achieved largely independently of the shelterin component TRF2. Similar findings have been obtained independently from parallel studies from the Lazzerini-Denchi laboratory (Markiewicz-Potoczny et al. 2020). While TRF2 localizes to ESC telomeres as part of the shelterin complex, ESCs without TRF2 show a severely attenuated telomeric DDR and do not undergo telomeric NHEJ. Consistently, ESCs can proliferate without TRF2, apparently indefinitely. However, end protection in ESCs is still dependent on the shelterin complex, as depletion of the entire shelterin complex from ESC telomeres leads to ATM activation, NHEJ-dependent telomere end-to-end fusions and rapid cell death. While multiple pluripotent lineages, including ESCs and epiblast stem cells (EpiSCs) apparently possess this largely TRF2-independent end protection mechanism, the differentiation of TRF2-null ESCs leads to a dramatic loss of end protection, robust activation of ATM and NHEJ at telomeres and cell death at the point that these cells exit the pluripotent state. Thus, in the context of early development, this alternate end protection mechanism is apparently restricted to the pluripotent state and TRF2 becomes essential for chromosome end protection when cells exit pluripotency upon differentiation. Finally, together with the Cesare laboratory (Ruis et al. 2020), we also observed that ESCs possess t-loops with a similar frequency to somatic cells, but unlike somatic cells, the formation of these loops is not dependent on TRF2.

This work, and other recent studies, cast new light on the t-loop model of chromosome end protection. In this perspective, we introduce shelterin and the mechanisms of ATM activation and NHEJ at telomeres, before discussing the following questions: How are t-loops proposed to protect chromosome ends and what is the evidence for this model? Can other models explain how TRF2 mediates end protection? Could t-loops be pathological structures? How is end protection achieved in pluripotent cells? What do the insights into telomere end protection in pluripotent cells mean for the t-loop model of end protection? Why might different cell states have evolved different mechanisms of end protection? Finally, we offer support for an updated t-loop model of end protection, suggesting that the data is supportive of a critical role for t-loops in protecting chromosome ends from NHEJ and ATM activation, but that other mechanisms are involved. Finally, we propose that t-loops are likely dynamic, rather than static, structures.

## The shelterin complex

Telomeric DNA is bound by specialized sets of proteins, whose composition, structure and function has diverged across species. In mammalian cells, six *bona fide* proteins specifically associate with telomeres in a complex termed shelterin (de Lange 2005): TRF1 (telomeric repeat-binding factor 1, also known as TERF1), TRF2 (telomeric repeat-binding factor 2, also known as TERF2), RAP1 (TERF2-interacting protein, also known as TERF2IP), TIN2 (TRF1-interacting nuclear factor 2, also known as TINF2), TPP1 (adrenocortical dysplasia protein homolog, also known as ACD), and POT1 (protection of telomeres 1) (Fig. 1B). Although a complete atomic-level structure of the shelterin complex is currently lacking, how the shelterin components interact with each other and with telomeric DNA, recruit other proteins to telomeres and mediate end protection is generally well-known. The shelterin complex components TRF1 and TRF2 bind with high (nanomolar) affinity to double stranded telomeric TTAGGG repeats via their Myb domains (Chong et al. 1995; Broccoli et al. 1997; Smogorzewska et al. 2000). TIN2 interacts with TRF1, TRF2 and TPP1, bridging these three proteins, while TPP1 interacts with POT1 (O'Connor et al. 2006; Hockemeyer et al. 2007; Wang et al. 2007; Kibe et al. 2010; Takai et al. 2011; Hu et al. 2017), enabling POT1 to coat the 3′ single-stranded G overhang by virtue of its oligonucleotide/oligosaccharide-binding (OB) folds (Kim et al. 1999; Baumann and Cech 2001; Baumann et al. 2002; Loayza and De Lange 2003; Lei et al. 2004; O'Connor et al. 2006; Hu et al. 2017). Rodents express two POT1 paralogs that emerged via gene duplication (POT1a and POT1b) and are structurally similar, yet functionally divergent (Hockemeyer et al. 2006; Wu et al. 2006). Finally, TRF2 interacts with RAP1, the most evolutionarily conserved component of shelterin (Li et al. 2000; Li and de Lange 2003). RAP1 has nontelomeric functions: It impacts nuclear factor κB (NF-κB) signaling (Teo et al. 2010), regulates transcription (Martinez et al. 2010, 2013), and is the only nonessential component of shelterin; knockout of any other component leads to cell and organismal inviability (Karlseder et al. 2003; Chiang et al. 2004; Celli and de Lange 2005).

Shelterin is thought to be expressed, localized to telomeres and responsible for end protection in all known mammalian systems (de Lange 2005, 2010, 2018; Sfeir and de Lange 2012). As the only complex that binds telomeres with a high affinity and sequence specificity, interactions with shelterin components represent the primary means through which other proteins and complexes are recruited to telomeres. These other proteins, which include nucleases, helicases, DNA damage factors and DNA replication proteins, act as shelterin cofactors, assisting shelterin in execution of end protection and telomere replication and extension (Oganesian and Karlseder 2009; de Lange 2018). They perform important functions including unwinding telomere secondary structures to enable passage of the replication fork (RTEL1 and BLM), resolving telomere secondary structures that could not be unwound (SLX1/4), generating 3′ G overhangs of an appropriate length for end protection (CST complex, APOLLO, and EXOI) and processing telomere ends (MRN complex, KU70/80) (d'Adda di Fagagna et al. 2001; Dimitrova and de Lange 2009; Sfeir et al. 2009; Wu et al. 2010, 2012; Ye et al. 2010; Vannier et al. 2012, 2013; Zimmermann et al. 2014). The shelterin complex both possesses its own intrinsic functions and co-opts the functions of these various cofactors to maintain end protection. Emerging data suggests these activities are tightly regulated throughout the cell cycle (Sarek et al. 2019).

Since TRF1 and TRF2 are the only proteins to bind telomeres with a high affinity and sequence specificity, codepletion of both TRF1 and TRF2 leads to “shelterin-free” telomeres that lose the end protective functions of shelterin and its cofactors. This reveals the full scope of the telomere end protection problem (Sfeir and de Lange 2012). Telomeres lacking shelterin are subject to the response of at least seven independent DDR pathways, any of which could cause gross genome instability if inappropriately activated at telomeres. The basic mechanisms through which shelterin and its cofactors inhibit these DDR pathways to achieve end protection are now generally understood (Fig. 1C,E; Palm and de Lange 2008; Sfeir et al. 2009; de Lange 2010, 2018; Kim et al. 2017). However, one topic that has remained controversial has been the protection of chromosome ends from the ATM and NHEJ DSB pathways by the shelterin component TRF2.

## The activation of ATM and NHEJ at DNA double-strand breaks

**Figure 2.**
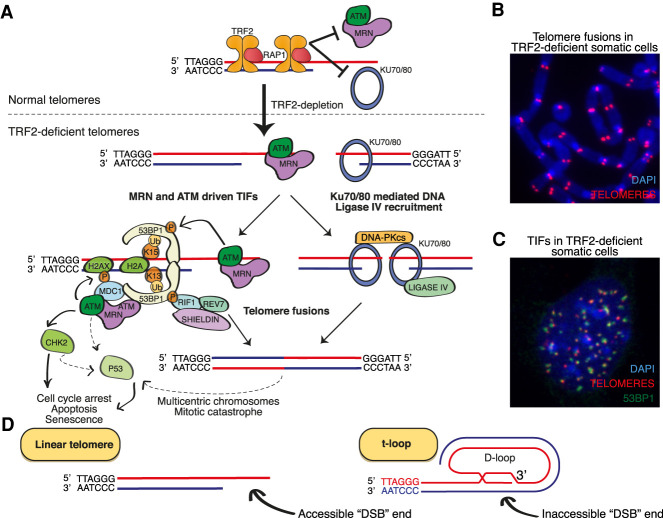
TRF2 prevents ATM and NHEJ at mammalian telomeres. (*A*) TRF2 inhibits the localization of the MRE11, RAD50, and NBS1 (MRN) and KU70/80 DNA double-strand break (DSB) sensors to chromosome ends. In somatic cells, TRF2 depletion deprotects telomeres leading to MRN and KU70/80 binding to telomeres. KU70/80 facilitates ligase IV-mediated NHEJ, leading to telomere fusions. Concurrently, MRN recruits ATM to chromosome ends, triggering a chain of protein modification and positive feedback loops that culminate in stable recruitment of MRN, ATM, MDC1, 53BP1, and the 53BP1 effectors RIF1, REV7, and Shieldin to telomeres, where they promote NHEJ. ATM also phosphorylates and activates CHK2 and P53, amongst other targets, leading to checkpoint arrest, apoptosis and senescence. (*B*,*C*) The removal of TRF2 from somatic cells leads to abundant telomere end-to-end fusions, visible in metaphase spreads, and telomeric dysfunction-induced foci (TIFs), which represent telomeres bound by the DNA repair proteins mentioned in *A*. (*D*) Linear telomeres have accessible ends that resemble “DSBs,” but telomere ends within looped (t-loop) conformations would remain inaccessible to, and hence protected from, DSB sensors.

DSBs are highly toxic lesions that can be repaired via three independent mechanisms: homology-directed repair (HDR, also known as HR), nonhomologous end joining (NHEJ), and microhomology-mediated repair (MMEJ, also known as alt-NHEJ) (Jackson and Bartek 2009; Chapman et al. 2012; Panier and Boulton 2014). The activation of HR and alt-NHEJ at telomeres has been extensively reviewed elsewhere (Doksani and de Lange 2014; de Lange 2018). DSBs are recognized by two parallel DSB end sensor complexes—KU70/80 and MRN—that associate with DSB ends within seconds of their formation (Fig. 2A; Uziel et al. 2003). Once bound to DSB ends, MRN recruits and activates the apical DDR signaling kinase Ataxia telangiectasia mutated (ATM) (for review, see Paull 2015). NBS1 and RAD50 both bind ATM directly, facilitating its recruitment to DSBs, while RAD50 promotes short distance (15 bp) unwinding of the DSB end, producing an optimal end for ATM activation (Cannon et al. 2013) and MRN acts as a cofactor for ATM activation (Paull 2015). ATM is autoinhibited in its typical dimeric form (Lee and Paull 2005; 2007), but when recruited to DSB ends by MRN, ATM monomerizes, becomes active and undergoes autophosphorylation on Ser1981. ATM activation thus requires both MRN and accessible DSB ends. Once activated, ATM coordinates the local and cellular response to DSBs, by phosphorylating in excess of 200 protein targets that promote lesion repair and cell cycle arrest to provide time for repair to ensue (Matsuoka et al. 2007; Jackson and Bartek 2009). Important ATM targets include: checkpoint kinase 2 (CHK2), which inhibits CDKs to stall cell cycling at the G1/S and G2/M transitions (Chehab et al. 2000; Matsuoka et al. 2000); P53, which coordinates both transient cell cycle arrest and (if P53 activation is prolonged) senescence or cell death (Shieh et al. 1997; Banin et al. 1998; Meek 2004; Roos et al. 2016); P53-binding protein 1 (53BP1) (Adams and Carpenter 2006; Chapman et al. 2013); the histone variant H2AX, which is phosphorylated on Ser139 to generate γH2AX (Burma et al. 2001).

Once generated by ATM at DSBs, γH2AX binds MDC1. MDC1 binds MRN, which in turn recruits and activates additional ATM at the DSB, producing a feed-forward loop that results in focal accumulation of γH2AX (Lukas et al. 2011). This facilitates a cascade of protein recruitment to, and ubiquitylation of, chromatin surrounding DNA breaks, involving the RING finger 8 (RNF8), RING finger 168 (RNF168) E3 Ubiquitin ligases and their E2 ligase UBC13 (Huen et al. 2007; Kolas et al. 2007; Mailand et al. 2007; Doil et al. 2009; Peuscher and Jacobs 2011; Mattiroli et al. 2012). This ubiquitylation enables the stable recruitment of additional repair factors to DSBs, including 53BP1, which binds to H2A ubiquitylated by RNF168 on Lys15 (Anderson et al. 2001; Bekker-Jensen et al. 2005). 53BP1 then acts as a scaffold with a range of protein-protein interaction sites that enable the recruitment of other factors involved in DSB repair to the lesion (Mirman and de Lange 2020). 53BP1 is itself phosphorylated by ATM on multiple N-terminal SQ/TQ sites; this phosphorylation enables the binding of 53BP1 to cofactors including the NHEJ mediator RIF1, with RIF1 then recruiting the Shieldin complex to DSBs (Chapman et al. 2013; Boersma et al. 2015; Xu et al. 2015; Dev et al. 2018; Ghezraoui et al. 2018; Gupta et al. 2018; Mirman et al. 2018; Noordermeer et al. 2018).

Unlike MRN, which is involved in both NHEJ and HR, the KU70/80 DNA end sensor is a specific component of classical NHEJ-mediated DSB repair (Weterings and van Gent 2004; Weterings and Chen 2008). KU70/80 forms a ring that binds DNA ends with a remarkable affinity (binding constant of 2 × 10^9^ M^−1^), is highly abundant within most cells (∼ 500,000 molecules per cell) and binds DSB ends in a sequence independent manner (Blier et al. 1993; Downs and Jackson 2004; Davis and Chen 2013). Once bound to DSB ends, KU70/80 serves as a scaffold to recruit other factors involved in NHEJ, including DNA-PKcs, DNA ligase IV, XRCC4-like factor (XLF), X-ray cross-complementing protein 4 (XRCC4), and aprataxin and PNK-like factor (APLF) (Davis and Chen 2013; Davis et al. 2014). These factors process DSB ends, producing a blunt ended substrate suitable for DNA ligase IV-mediated ligation (Wilson et al. 1997). This contrasts with HR, which requires extensive resection of DSB to produce ssDNA suitable for RAD51 loading and the homology search that is essential for HR. Recent evidence suggests the central axis of the HR versus NHEJ pathway choice is the regulation of resection: Resection of DSBs favors HR, while the retention of relatively blunt ends favors NHEJ (Panier and Boulton 2014).

While the core NHEJ components are sufficient for NHEJ in vitro and at simple blunt-ended DSBs in G1, additional factors including ATM, 53BP1 and its cofactors RIF1 and Shieldin are required in many in vivo contexts, including at physiological DSBs generated during class switch recombination (CSR), DSBs within heterochromatin and at dysfunctional telomeres (Escribano-Díaz et al. 2013; Zimmermann et al. 2013; Xu et al. 2015; Ceccaldi et al. 2016; Mirman et al. 2018; Noordermeer et al. 2018; Mirman and de Lange 2020). 53BP1 promotes the three-dimensional motility of DNA breaks, facilitating synapsis of distal DNA ends, while Shieldin counteracts DNA end resection to promote NHEJ over HR at DSBs (Lottersberger et al. 2015; Dev et al. 2018; Ghezraoui et al. 2018; Mirman et al. 2018). Shieldin may achieve this by directly inhibiting DNA end resection, promoting CST/Polα/Primase-mediated fill-in of already resected DNA ends or via both pathways. Highly heterochromatic regions are also refractory to NHEJ (Goodarzi et al. 2008). At DSBs in such regions, 53BP1 amplifies MRE11-NBS1 accumulation, concentrating active ATM to facilitate repair through robust local phosphorylation of KAP1, which then promotes repair through chromatin relaxation (Noon et al. 2010). Thus, while not essential components of NHEJ *per se*, 53BP1 and its cofactors are essential for NHEJ in particular contexts. Since ATM is required for stable 53BP1, RIF1 and Shieldin recruitment to DSBs, NHEJ that relies on 53BP1 is also largely dependent on ATM.

## The importance of TRF2 for mammalian chromosome end protection

The efficient mechanisms that recognize and repair DSBs discussed above pose an obvious threat to eukaryotes with linear chromosomes; the ends of these chromosomes are ideal substrates for the binding of MRN and KU70/80 and hence the activation of ATM and NHEJ. Telomeres clearly solve this end protection problem; chromosome ends in mammalian cells do not constitutively activate ATM or undergo NHEJ. A series of seminal papers revealed that the removal of TRF2 from telomeres leads to chromosome end-to-end fusions, CHK2-mediated G2/M cell cycle arrest and p53-mediated senescence and cell death (van Steensel et al. 1998; Karlseder et al. 1999). These telomere fusions are dependent on the core NHEJ factors KU70/80, ligase IV, and DNA-PKcs; the ATM kinase and its DNA sensor MRN; and the NHEJ accessory factors 53BP1, RIF1, and Shieldin (Fig. 2A,B; Smogorzewska et al. 2002; Celli and de Lange 2005; Celli et al. 2006; Denchi and de Lange 2007; Dimitrova et al. 2008; Deng et al. 2009; Dimitrova and de Lange 2009; Chapman et al. 2013; Zimmermann et al. 2013; Ghezraoui et al. 2018). Like at bona fide DSBs, ATM is recruited to telomeres by MRN upon TRF2-loss, generating γH2AX and triggering the γH2AX/MDC1/MRN/ATM feed-forward mechanism that leads to stable recruitment of RNF8, RNF168, and 53BP1 and its cofactors RIF1 and Shieldin to telomeres (de Lange 2018). Once recruited to TRF2-null telomeres, 53BP1 cooperates with the LINC complex to promote their microtubule-dependent motility and, together with RIF1/Shieldin, counteracts excessive telomere resection to maintain overhangs of an appropriate length for NHEJ (Dimitrova et al. 2008; Lottersberger et al. 2015; Mirman et al. 2018). 53BP1, γH2AX and many of these other DSB repair factors can be readily detected at telomeres in TRF2-null cells via indirect immunofluorescence (IF) staining, where they form discrete foci termed telomere dysfunction induced foci (TIFs) (Fig. 2C; Takai et al. 2003). The accumulation of TIFs, CHK2-mediated cell cycle arrest, telomere fusions and p53 activation in TRF2-null cells is entirely dependent on ATM, suggesting this is the sole kinase responsible for coordinating the DDR at TRF2-null telomeres (Celli and de Lange 2005; Denchi and de Lange 2007). Likewise, the TIFs in TRF2-null cells are dependent on MRN, while these telomere fusions are dependent on both MRN and KU70/80 (Celli et al. 2006; Deng et al. 2009; Dimitrova and de Lange 2009).

Somewhat counterintuitively, multiple components of ATM and NHEJ signaling that contribute to NHEJ at TRF2-null telomeres are actually required to maintain complete chromosome end protection in TRF2-proficient cells. MRE11, NBS1, and ATM are specifically recruited to functional telomeres in G2 (Verdun et al. 2005; Verdun and Karlseder 2006), following telomere replication, while the loss of KU70, KU80, DNA-PKcs, or MRN is sufficient to induce a small number of telomere fusions in cells with functional TRF2, presumably via alt-NHEJ (d'Adda di Fagagna et al. 2001; Gilley et al. 2001; Gao et al. 2009; Rybanska-Spaeder et al. 2014). It seems likely that these proteins, which all possess DSB end processing functions, promote proper processing of chromosome ends to mediate end protection, perhaps assisting in the production of 3′ G overhangs suitable for t-loop formation. How these functions are coordinated remains unknown.

Thus, TRF2 is a crucial component of the mammalian solution to the end protection problem and prevents the activation of ATM and NHEJ at chromosome ends. Unlike at bona fide DSBs, NHEJ at TRF2-null telomeres does not require extensive end processing, but rather depends on endonucleolytic cleavage of the 3′ G overhang, possibly by ERCC1/XPF, as part of the ligation reaction (Zhu et al. 2003; Celli and de Lange 2005). The protection of chromosome ends from ATM and NHEJ by TRF2 has, until recently, been regarded as a universal feature of mammalian chromosome end protection. The depletion of TRF2 from somatic mouse cells and various human cell lines leads to telomeric ATM activation, NHEJ and is incompatible with viability (van Steensel et al. 1998; Karlseder et al. 1999; Kim et al. 2017). Likewise, Cre-lox mediated systems used to knockout TRF2 specifically in mouse epidermal, liver, and neural tissues in each case induces ATM activation and NHEJ at telomeres, which in tissues with cycling cells leads to cell death and loss of that tissue (Zhu et al. 2003; Lazzerini Denchi et al. 2006; Martinez et al. 2014; Lobanova et al. 2017). The question that remains is how TRF2 achieves this inhibition of NHEJ and ATM.

## The t-loop model of end protection

Microscopic interrogation of telomeres has revealed that mammalian telomeres often end in a looped structure, termed a telomere (t)-loop (Griffith et al. 1999; Doksani et al. 2013). It is proposed that this structure forms through the invasion of the 3′ G overhang into the upstream telomeric dsDNA, where it base-pairs with the C strand, displacing the G strand in this region. The observed loops vary in size from 1 to 20 kb, suggesting this invasion occurs in a positionally blind manner, with any telomeric dsDNA available for the invasion of the 3′ G overhang. Given telomeres share a single hexameric repetitive sequence, this is certainly plausible. The t-loop is an attractive structure to mediate end protection as it would sequester the extreme chromosome terminus, hiding the linear DNA end that would otherwise bind KU70/80 and MRN. Thus, t-loops would be expected to simultaneously block the initiation steps of both ATM activation and NHEJ. Since TRF2 blocks both these pathways at telomeres, a hypothesis quickly developed that TRF2 might mediate end protection primarily through promoting the formation and/or stabilization of these loops. In its simplest formulation, this is the t-loop model of end protection: The TRF2-dependent formation of t-loops at chromosome ends prevents their misidentification as DSBs and the activation of ATM and NHEJ (Fig. 2D).

Testing this model directly is challenging, but it makes many predictions that can be experimentally tested. If they are a key mediator of end protection, t-loops should be present at the majority of chromosome ends; t-loops should be present when protection from ATM and NHEJ is achieved, but t-loops should disappear when this end protection is lost; the unwinding of t-loops should induce ATM and/or NHEJ activation. Likewise, if TRF2 mediates the formation of these loops, t-loops should be present in cells with TRF2 but disappear when TRF2 is lost; TRF2 should possess some specific domain/mechanism that can promote the formation or stabilization of t-loops; the loss of this domain/mechanism should remove t-loops and induce ATM and NHEJ at telomeres. Finally, if t-loop formation is the central component of protection from ATM and NHEJ, t-loops alone should be sufficient to protect chromosome ends from ATM and NHEJ.

## T-loops exist and are lost concurrently with end protection

Obtaining an accurate estimate of the frequency of t-loops within a population of cells is a significant challenge. The two available techniques, EM and superresolution microscopy, use the same basic method to prepare telomeres for visualization. DNA is harvested and cross-linked via UV and Psoralen, preserving DNA secondary structures that arise in vivo. For EM visualization, DNA is incubated with frequent-cutter restriction endonucleases that exclusively digest nontelomeric DNA, freeing individual telomeres from the intervening DNA (Griffith et al. 1999). Telomeric DNA can then be purified from genomic DNA via a gel-filtration column and spread onto EM grids. For superresolution microscopy visualization, DNA extracts are spread onto slides and telomeric DNA is labeled with a sequence-specific telomere probe (Doksani et al. 2013). In each case, the macroscopic structure of cross-linked telomeric DNA is visualized, allowing an estimation of the frequency of telomeres with terminal loops to be made. EM detects loops at roughly 15%–40% of telomeres, while superresolution microscopy has produced estimates of 25%–35% looped telomeres in a diverse range of contexts, from human somatic and cancer cells and pluripotent and somatic mouse cells, amongst others, suggesting t-loops exist with similar frequencies across different mammalian organisms and cell types (Griffith et al. 1999; Doksani et al. 2013; Van Ly et al. 2018).

**Figure 3.**
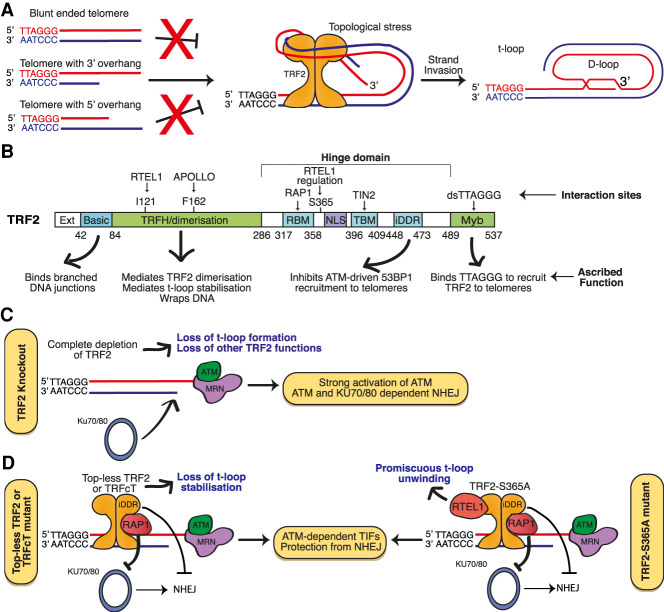
TRF2 promotes t-loop stabilization to protect chromosome ends. (*A*) T-loops require telomeric DNA possessing a 3′ overhang. TRF2 associates with the telomeric dsDNA sequence, wrapping 90 bp of DNA with various lysine/alanine residues in its TRFH domain, applying a topological stress that is proposed to promote the invasion of the 3′ overhang into the ds telomeric DNA. (*B*) Depiction of the multiple discrete domains of TRF2, their interactions and functions. (*C*) Complete depletion of TRF2 leads to loss of t-loops and t-loop independent functions of TRF2, leading to strong activation of ATM and ATM and NHEJ-dependent telomere fusions. (*D*) Mutants of TRF2 that are unable to form t-loops, or tethering of RTEL1 to TRF2 to promote promiscuous t-loop unwinding, leads to a reduction in t-loops and activation of ATM but not NHEJ. These telomeres that are compromised for t-loop stabilization therefore retain some protection from NHEJ that is lost when TRF2 is removed entirely. This t-loop independent protection from NHEJ is enacted, at least in part, by RAP1 and the TRF2-iDDR domain.

Could these loops simply be an artefact of the cross-linking and/or visualization process? Several pieces of evidence strongly dispute this notion. T-loops are never observed at both ends of a purified single telomere via EM, indicating that the “t-loop” objects being observed possess end specificity and are not simply the result of “sticky” DNA ends (Griffith et al. 1999). Moreover, t-loops can be observed without cross-linking, albeit at significantly lower frequencies (Griffith et al. 1999). Most compellingly, multiple studies have now shown that the depletion of TRF2 leads to a dramatic reduction in the frequency of t-loops when measured by superresolution microscopy, from 25%–35% to 5%–10% (Fig. 3A; Doksani et al. 2013; Benarroch-Popivker et al. 2016; Van Ly et al. 2018; Tomáška et al. 2020). This indicates that the structures observed as “t-loops” are specific products of TRF2 function and not an artefact of the telomere preparation and visualization process. Therefore, t-loops really do form at chromosome ends in vivo and they arise in a largely TRF2-dependent manner.

However, if cross-linking is incomplete, telomeres break during spreading or loops reside along the z-axis during imaging, looped telomeres will be misidentified as linear. Moreover, superresolution microscopy techniques have maximal resolutions of 20 nm (STORM) and 140 nm (Airyscan), corresponding to ∼ 70 bp and ∼ 500 bp of dsDNA respectively. These maximal technical resolutions are rarely reached in practice. Small t-loop structures would thus be indistinguishable from linear telomeres in these analyses. Similarly, linear telomeres that crossed themselves during spreading might be misidentified as looped. Collectively therefore, the false-negative rate and, to a lesser extent false-positive rate, for t-loop identification is likely very high, meaning these techniques can only be used to estimate relative t-loop frequency and not the *absolute* frequency of looped telomeres. These techniques demonstrate that t-loops exist, but leave open the possibility that anything from 30% to 100% of telomeres reside in t-loops within a population of cells.

## The mechanism of TRF2-mediated t-loop stabilization

As mentioned above, the presence of t-loops within cells is largely dependent on TRF2 (Fig. 3A). TRF2 is expressed as two functionally indistinct proteins, distinguished by the addition of a 42-amino-acid N-terminal extension, and has a series of highly conserved domains, including: the TRFH domain, which enables the homodimerization of TRF2; a C-terminal Myb domain, which allows TRF2 to bind ds telomere (TTAGGG) repeats directly; the N-terminal basic domain, which binds to DNA junctions; and the Hinge domain, which has been implicated in mediating various protein–protein interactions (Fig. 3B; de Lange 2010; Okamoto et al. 2013). TRF2, but not TRF1, can promote the formation of t-loops when added to model telomeric DNA in vitro. This is dependent on the presence of ds TTAGGG repeats and a 3′ G overhang; termini with 5′ overhangs, blunt ends, or 3′ termini with nontelomeric sequences at the ds/ss junction cannot form loops in vitro, and involve the binding of TRF2 near the ds/ss junction point (Griffith et al. 1999; Stansel et al. 2001). Thus, TRF2 likely promotes invasion of the 3′ G overhang into the ds telomeric DNA, as predicted by the t-loop model. This is analogous to the formation of D-loops during RAD51-mediated HR, in which RAD51 coats the ssDNA molecule and catalyzes its invasion into a homologous template. However, unlike RAD51, TRF2 lacks enzymatic domains or ATP-hydrolyzing activity so it cannot actively catalyze the invasion event that leads to t-loop formation. It therefore seems reasonable to propose that TRF2 stabilizes t-loops rather than directly mediates their formation.

Insights into how TRF2 might stabilize t-loops have come from genetic studies with various mutants of TRF2. The TRFH domain contains a series of exposed Lysines and Arginines that interact with DNA in a sequence-independent manner. This allows dimeric TRF2 to wrap 90 bp of telomeric DNA around itself, promoting DNA condensation and exerting a topological stress onto the dsDNA (Amiard et al. 2007; Poulet et al. 2012; Benarroch-Popivker et al. 2016). A TRF2 mutant in which these lysines and arginines are substituted for alanines, named Top-less TRF2, can no longer condense telomeric DNA. Cells expressing Top-less TRF2, and cells expressing a TRFcT mutant lacking the entire TRFH domain of TRF2, possess significantly fewer t-loops than cells expressing wild type TRF2 (Benarroch-Popivker et al. 2016; Van Ly et al. 2018). Indeed, the level of t-loops in these cells is indistinguishable from cells completely lacking TRF2. Thus, the TRFH domain of TRF2 is necessary for t-loop formation, suggesting TRF2 exerts a topological stress onto ds telomeric DNA, entropically favoring the product of this invasion over individual linear telomeres (Okamoto et al. 2013; Benarroch-Popivker et al. 2016; Van Ly et al. 2018).

## T-loops protect chromosome ends from ATM activation and NHEJ but are not solely responsible for this

T-loops exist at some chromosome ends and arise in a manner that likely depends on TRF2 exerting topological stress on dsDNA to facilitate 3′ G overhang invasion or stabilize the t-loop three-way junction. What is the impact of the t-loop on protection from ATM and NHEJ? The formation of t-loops depends on both TRF2 and the 3′ G overhang. Complete removal of TRF2, as discussed, leads to dramatically fewer t-loops and robust activation of NHEJ and ATM at telomeres, resulting in inviability (Fig. 2; Doksani et al. 2013). However, it is impossible to say from these data alone whether the loss of t-loops is responsible for the activation of ATM and NHEJ—TRF2 could have other functions that are important for end protection that are also lost coincidentally with t-loops in these experiments (Arnoult and Karlseder 2015).

Due to the nature of semi-conservative DNA replication, the 3′ G overhang is retained at lagging telomeres but must be generated de novo at each leading end telomere after telomere replication. This is achieved by the TRF2-mediated recruitment of the exonuclease APOLLO to leading end telomeres. The knockout of APOLLO, or abrogation of the APOLLO-TRF2 interaction through a TRF2-F162 mutant, prevents timely formation of leading end G overhangs and, since t-loops require an overhang, this necessarily restricts t-loop formation at leading end telomeres (van Overbeek and de Lange 2006; Lam et al. 2010; Wu et al. 2010). Consistent with the proposed protective role of t-loops, cells lacking APOLLO or expressing an APOLLO-binding deficient TRF2-F162A mutant show robust activation of ATM and NHEJ specifically at leading end telomeres (Wu et al. 2010). Likewise, cells expressing the Top-less or TRFcT mutants show a similar reduction in t-loop frequencies to cells lacking TRF2 and show robust telomeric activation of ATM. However, the linear telomeres formed in cells expressing Top-less or TRFcT mutants are still protected from NHEJ (Benarroch-Popivker et al. 2016; Van Ly et al. 2018). Thus, linear telomeres are still protected from NHEJ by TRF2 mutants that cannot form t-loops, suggesting TRF2 protects telomeres from NHEJ independently of t-loop formation.

Consistent with this notion, time course studies of t-loops and the DDR upon removal of TRF2 reveal that ATM activation coincides with the loss of t-loops and precedes telomeric NHEJ by 12–24 h (Van Ly et al. 2018). Another recent study investigated the impact of reduced levels of t-loops in the presence of functional TRF2. The TRF2-S365A mutant constitutively binds the t-loop unwinding helicase regulator of telomere length 1 (RTEL1), tethering RTEL1 to telomeres. Expression of TRF2-S365A induces promiscuous t-loop unwinding, producing somatic cells with fewer t-loops but otherwise fully functional TRF2 (Sarek et al. 2019). The linear telomeres resulting from excessive RTEL1 t-loop unwinding induce an ATM-dependent DDR but do not activate NHEJ. This confirms that t-loops are required to fully repress ATM activation at telomeres and that TRF2 is able to protect telomeres from NHEJ when ATM is activated at linear telomeres. This ATM-activated but NHEJ-repressed state has been proposed to represent an “intermediate state” of telomere end protection and has now been observed in multiple scenarios. Telomeres in cells blocked in mitosis activate ATM but not NHEJ (Cesare et al. 2009, 2013; Van Ly et al. 2018); telomeres in cells with partially reduced TRF2 expression activate ATM without strongly activating NHEJ (Cesare et al. 2009, 2013; Van Ly et al. 2018); critically short telomeres produced through aging activate ATM but very rarely undergo NHEJ (Hewitt et al. 2012; Kaul et al. 2012; Hayashi et al. 2015); cells expressing Top-less or TRFcT mutants (which lack t-loops) robustly activate telomeric ATM but not NHEJ (Okamoto et al. 2013; Benarroch-Popivker et al. 2016; Van Ly et al. 2018); telomeres engineered to possess TRF2 but fewer t-loops via the TRF2-S365A mutant activate ATM but not NHEJ (Fig. 3C; Sarek et al. 2019). Thus, telomeres can activate ATM without NHEJ and do so when the frequency of t-loops is reduced but some TRF2 functionality remains present. This suggests that t-loops protect telomeres from ATM activation, but that t-loops are not essential for protection from NHEJ and hence ATM and NHEJ activation can be uncoupled at telomeres.

## Alternative mechanisms of TRF2-mediated end protection

While t-loops are observed in organisms as diverse as vertebrates (Griffith et al. 1999), plants (Cesare et al. 2003, 2008) and trypanosomes (Munoz-Jordan et al. 2001), they are not universal (Tomáška et al. 2020). Chromosome end protection is maintained without t-loops in the small linear DNA fragments found in the macronuclei of hypotrichous ciliates, where end protection relies on a proteinaceous cap (Gottschling and Zakian 1986), and in dipteran insects, which lack G-rich telomeric sequences entirely and instead cap their chromosome ends with long retrotransposons (Young et al. 1983; Biessmann and Mason 1997). Thus, end protection can be achieved without t-loops in certain species. Indeed, mammalian cells expressing TRF2-S365A, TRFcT or Top-less (which have reduced t-loops) robustly activate telomeric ATM but do not robustly activate NHEJ. Therefore, in mammals t-loop stabilization cannot be the sole means through which TRF2 mediates end protection.

One proposal is that TRF2 contributes to the compaction of telomeric chromatin through a complex network of interactions between shelterin subunits and telomeric DNA. In this model, the tightly compacted telomeric chromatin would prevent KU70/80 and MRN from accessing the end of the chromosome, removing the requirement for a t-loop. Consistent with this possibility, the removal of individual shelterin subunits, including TRF2, or mutations that abrogate shelterin assembly, were suggested to induce a 10-fold increase in telomere volume, coincident with the activation of telomeric ATM signaling (Bandaria et al. 2016). However, multiple independent studies, in mouse and human cells, have failed to recapitulate the essential phenotype predicted by this model, namely that the removal of TRF2, or the entire shelterin complex, from telomeres should induce three-dimensional decompaction, concomitant with DDR activation (Timashev et al. 2017; Vancevska et al. 2017). One possible explanation for this disparity is that dysfunctional telomeres can become clustered, giving the misleading impression of telomeric decompaction (Dimitrova et al. 2008). Indeed, if chromatin compaction is responsible for the inhibition of ATM at telomeres, this should prevent ATM activation at breaks within the telomere. However, telomere-internal DSBs generated by TRF1-FOK1 induce the robust activation of ATM (Doksani and de Lange 2016). Shelterin-mediated telomere compaction is therefore insufficient to explain the repression of ATM and NHEJ at telomeres.

Another alternate proposal is that TRF2 might inhibit DDR factors directly. TRF2 interacts with ATM, MRN and KU70/80, providing multiple avenues through which this could occur (Song et al. 2000; Karlseder et al. 2004; Okamoto et al. 2013). Indeed, TRF2 has been proposed to bind to chromosomal DSBs, where it might influence both ATM activation and repair, and TRF2 contains multiple canonical S/T-Q ATM phosphosites, suggesting ATM could regulate TRF2 function (Bradshaw et al. 2005; Matsuoka et al. 2007). A short 30-amino-acid inhibitor of the DDR (iDDR) domain has been found within the TRF2 Hinge domain (Okamoto et al. 2013). TRF2-iDDR interacts with the MRN complex and, via MRN, recruits the deubiquitination enzyme BRCA1–BRCA2-containing complex 3 (BRCC3), which prevents H2A polyubiquitination–dependent recruitment of RNF168, and ubiquitin protein ligase 5 (UBR5), an enzyme that mediates degradation of RNF168, to telomeres (Okamoto et al. 2013). The expression of TRF1 fused to the TRF2-iDDR domain is sufficient to reduce, but not abolish, NHEJ at TRF2-null telomeres, without impacting ATM activation. Thus, TRF2-iDDR is proposed to repress NHEJ downstream from ATM activation, by limiting the accumulation of RNF168, and thereby 53BP1, at telomeres. This provides at least one t-loop independent mechanism through which TRF2 can repress NHEJ.

TRF2 has also been proposed to limit telomeric NHEJ through an undefined function of its interacting partner RAP1. Unlike other shelterin components, RAP1 is not essential for viability or end protection; cells lacking RAP1 show no telomere dysfunction phenotypes and do not activate either ATM or NHEJ (Sfeir et al. 2010). However, the tethering of RAP1 to TRF2-deficient telomeres reduces telomeric NHEJ (Sarthy et al. 2009), TRF2/RAP1, but not TRF2 alone, can inhibit NHEJ at telomeric substrates in vitro (Bae and Baumann 2007) and the depletion of RAP1 from cells expressing the Top-less TRF2 mutant (which lack t-loops and activate ATM without concomitant NHEJ) induces telomeric NHEJ (Benarroch-Popivker et al. 2016). Thus, while RAP1 is not essential for end protection per se, TRF2 appears to recruit RAP1 to inhibit NHEJ at linear telomeres independently of ATM activation.

## T-loops as pathological structures

**Figure 4.**
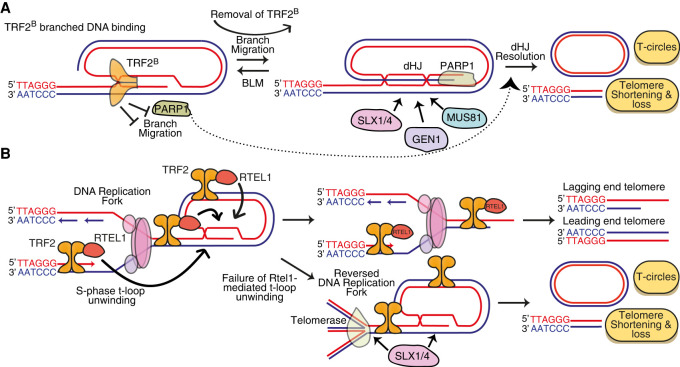
TRF2 coordinates t-loop transactions to ensure they remain protective, not pathological, structures. (*A*) The TRF2 basic domain (TRF2^B^) binds to branched DNA junctions, including those found at the base of the t-loop, and prevents their migration from three-way into four-way junctions, which are idealized substrates for double Holliday junction (dHJ) resolvases, including SLX1/4, GEN1 and MUS81. TRF2^B^ also inhibits PARP1 activity at telomeres. Collectively, these TRF2^B^ functions prevent dHJ resolution and hence telomere shorterning and T-circle formation. (*B*) TRF2 recruits RTEL1 to telomeres in S phase through a phosphorylation-regulated interaction between TRF2-S365 and RTEL1. RTEL1 then unwinds t-loops, enabling the passage of the replication fork through this DNA Secondary structure and complete telomere replication. Failure of RTEL1-mediated t-loop unwinding leads to replication fork reversal within the telomere and cleavage of either the reversed fork or residual t-loops themselves by SLX1/4, leading to potentially catastrophic telomere shorterning and T-circle formation.

While proposed to be protective structures, t-loops can actually pose a threat to end protection, leading to the alternate notion that t-loops might be pathological, not protective, structures. If a t-loop undergoes branch migration to form a double Holliday junction (dHJ) (Fig. 4A), it becomes an optimal substrate for cleavage by HJ resolvases, including MUS81, SLX1/SLX4, EMI1 and/or GEN1 (Wyatt and West 2014). This cleavage results in large telomere deletions and Telomere Circle (TC) formation and is promoted by poly(ADP-ribose) polymerase (PARP1), which can bind to 5′ ss-dsDNA junctions, and repressed by the basic domain of TRF2 (TRF2^B^) (Wang et al. 2004; Saint-Léger et al. 2014; Schmutz et al. 2017). TRF2^B^ binds to branched DNA junctions in vitro and in vivo, while deletion of TRF2^B^ induces telomeric accumulation of PARP1 and TC formation via t-loop cleavage (Fouché et al. 2006; Poulet et al. 2012; Schmutz et al. 2017). TRF2^B^ can be functionally replaced with bacterial branched DNA-binding domains, suggesting the key function of TRF2^B^ is to bind the DNA junction at the base of the t-loop, blocking branch migration at the base of the t-loop to prevent dHJ formation (Schmutz et al. 2017). The TRF2 basic domain also directly blocks the telomeric recruitment of PARP1 (Rai et al. 2016). The BLM helicase, which dissolves dHJs, represses the cleavage of t-loops in the absence of the TRF2 basic domain, presumably by mediating the reversion of telomeric dHJs into three-way junctions (Fig. 4A).

Like other DNA secondary structures, t-loops can stall the replication fork and therefore require unwinding during S phase (Mirkin and Mirkin 2007; Vannier et al. 2012, 2013). This is achieved by the RTEL1 helicase, which is recruited to telomeres in S phase when TRF2-S365 is dephosphorylated by PP6R3, enabling TRF2 to interact with and recruit RTEL1 to telomeres (Vannier et al. 2012, 2013; Sarek et al. 2015a,b; Sarek et al. 2019). This provides a brief window for RTEL1 to unwind t-loops, facilitating the passage of the DNA replication machinery. RTEL1 unwinds D-loops in vitro, while tethering of RTEL1 to telomeres via a TRF2-S365A mutant reduces t-loop levels in vivo, suggesting RTEL1 unwinds t-loops directly (Barber et al. 2008; Vannier et al. 2012). However, RTEL1 also unwinds telomeric G-quadruplex structures and possibly RNA-DNA hybrids (R-loops), although RTEL1 is recruited to these secondary structures through an interaction with PCNA, not with TRF2 (Vannier et al. 2012; Björkman et al. 2020; Wu et al. 2020). Therefore, RTEL1 might also impact t-loops indirectly, by unwinding secondary structures that alter topological stress within the telomere. Loss of RTEL1, or abrogation of TRF2-mediated RTEL1 recruitment to telomeres, leads to replication fork stalling and reversal, in a manner dependent on telomerase and the fork reversal machinery (Margalef et al. 2018). These reversed forks and/or t-loops themselves are then cleaved by SLX1/SLX4, inducing TC formation and telomere shortening (Fig. 4B; Vannier et al. 2012).

Progressive telomere shortening in cells that fail to unwind t-loops, or in cells with unrestrained dHJ formation, eventually generates critically short telomeres that are unable to mediate end protection. This is exemplified by mutations in RTEL1, which cause Hoyeraal–Hreidarsson syndrome (HHS), a syndrome typified by very short, heterogenous telomeres that drive senescence, premature aging and cancer (Sarek et al. 2015). Thus, when t-loops persist throughout telomeric DNA replication or are converted into dHJs, they become pathological structures. Mammals have evolved an elegant solution to this potential problem: The same protein that mediates t-loop formation (TRF2) ensures the t-loop does not become toxic by coordinating timely t-loop unwinding, through RTEL1, and by blocking PARP1 recruitment and branch migration at t-loops, through the TRF2^B^ domain. Thus, TRF2 ensures t-loops are protective, not pathological, structures in normal conditions.

## End protection in ESCs

The absolute dependency of end protection on TRF2 has been confirmed in numerous scenarios, including somatic mouse and human cells and in mouse liver, skin and neuronal compartments in vivo (van Steensel et al. 1998; Karlseder et al. 1999; Lazzerini Denchi et al. 2006; Martinez et al. 2014; Kim et al. 2017; Lobanova et al. 2017). In all of these contexts, the loss of TRF2 leads to robust ATM activation and rapid accumulation of telomere fusions as a result of NHEJ. On this basis, TRF2 was assumed to have a universal role in end protection throughout mammalian development. Surprisingly, two recent studies have revealed that chromosome end protection in the pluripotent stages of early development occurs largely independently of TRF2 (Markiewicz-Potoczny et al. 2020; Ruis et al. 2020). While these studies confirmed previous findings (that TRF2-depletion in somatic cells leads to ATM activation, NHEJ, cell cycle arrest and cell death), they establish that the loss of TRF2 from mouse ESCs leads to a mild activation of ATM but with no evidence of significant telomeric NHEJ, CHK2 activation, cell cycle arrest, or cell death. However, the depletion of the entire shelterin complex from ESC telomeres leads to robust telomeric activation of ATM and NHEJ, CHK2 activation, G2/M-phase cell cycle arrest and rapid loss of viability (Ruis et al. 2020). Thus, in ESCs the inhibition of ATM and NHEJ at telomeres is achieved in a largely TRF2-independent, but shelterin-dependent, manner. While this largely TRF2-independent end protection is apparently present in multiple distinct pluripotent states, including ESCs, EpiSCs, and pluripotent cells in murine E3.5 embryos, differentiation of TRF2-null ESCs rapidly leads to robust telomeric ATM activation, telomere fusions by NHEJ and cell death at the point that TRF2-null cells exit from pluripotency (Markiewicz-Potoczny et al. 2020; Ruis et al. 2020). Thus, the TRF2-independent protection of telomeres from ATM and NHEJ is, in the context of early development, uniquely restricted to the pluripotent stage.

**Figure 5.**
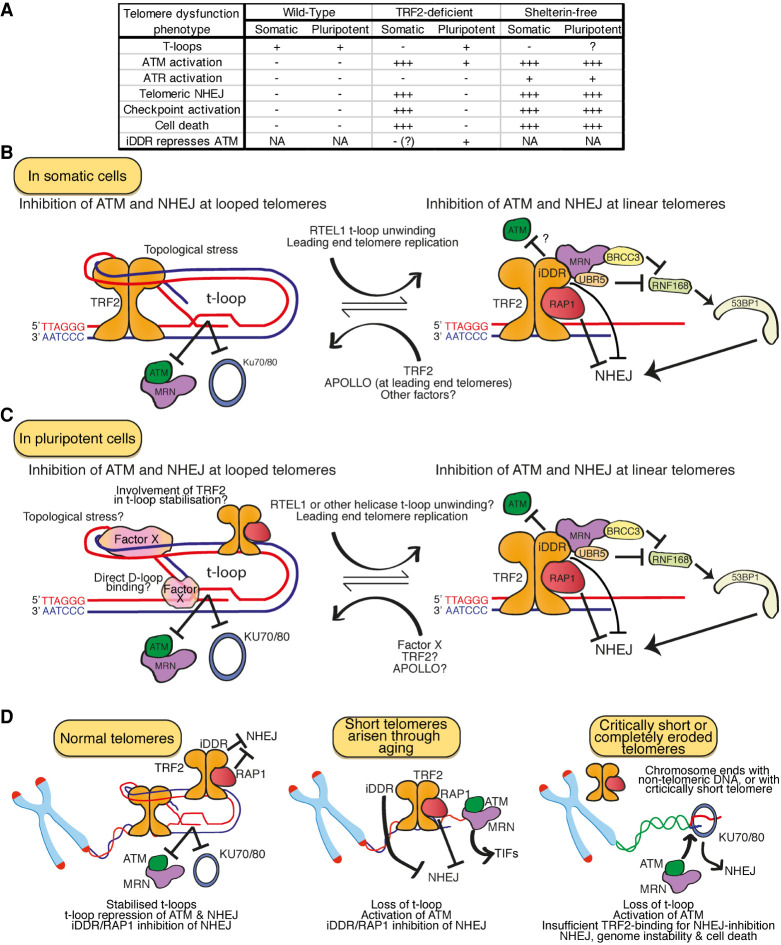
Insights from pluripotent telomere end protection. (*A*) Table of the phenotypes observed in wild-type, TRF2-deficient and shelterin-free somatic and pluripotent cells. Most notably, while somatic cells lacking TRF2 undergo strong ATM activation, checkpoint activation and cell death and display large amounts of telomeric NHEJ, concomitantly with the loss of t-loops, pluripotent cells lacking TRF2 retain the same level of t-loops as both wild type somatic and pluripotent cells, show an attenuated ATM activation and do not display telomeric NHEJ, checkpoint activation or cell death. (*B*) In somatic cells, TRF2 stabilizes t-loops by applying topological stress to the telomeric dsDNA. Telomeres within t-loops remain protected from ATM and NHEJ since their ends are hidden from the MRN and KU70/80 DNA end sensors. RTEL1 unwinds t-loops in S phase, while leading end telomeres are blunt after replication so must also be linear, creating a requirement for the protection of linear telomeres from ATM and NHEJ. TRF2 protects linear telomeres through its inhibitor of the DNA Damage Response (iDDR) domain and by recruiting RAP1 to telomeres, both of which repress NHEJ. (*C*) In pluripotent cells, we propose that some protein performs a functionally homologous role to TRF2, here termed “factor X,” and promotes the stabilization and/or formation of t-loops, to inhibit NHEJ and ATM at telomeres. Whether TRF2 also promotes t-loop stabilization, whether RTEL1 unwinds t-loops and APOLLO mediates leading end G-overhang formation in wild type ESCs remains unclear, but since RTEL1 and APOLLO are usually recruited to telomeres by TRF2, either alternative mechanisms to recruit these factors, or alternative proteins to perform these functions, are required. (*D*) At normal somatic telomeres, TRF2 coordinates the inhibition of ATM and NHEJ, in part through stabilizing t-loops, which must have a minimum length requirement. During aging, telomere length progressively shortens, producing short telomeres. One possibility to explain ATM-positive but NHEJ-resistant aged telomeres is that they are too short to form t-loops but long enough to bind TRF2 and retain t-loop-independent end protection functions. Critically short telomeres that produce chromosome ends without TRF2 binding lose these activities, allowing telomere fusions that precipitate genome instability and cell death.

Given the inextricable link between TRF2, end protection and t-loop formation in somatic cells, it was important to consider the status of t-loops in ESCs. The Boulton and Cesare laboratories (Ruis et al. 2020) visualized telomeric secondary structures in ESCs via superresolution microscopy. This revealed that ESCs possess t-loops with similar size and frequency (∼30% of telomeres) to somatic cells. However, unlike somatic cells where these loops are lost upon TRF2 depletion, in ESCs the frequency and size of t-loops is unaffected by the loss of TRF2, suggesting that in ESCs the formation and stabilization of t-loops occurs in a TRF2-independent manner (Fig. 5A; Ruis et al. 2020). Collectively, these unexpected discoveries prompt a series of questions and also cast new light on the existing paradigms of telomere end protection, both discussed below.

## Insights regarding chromosome end protection from ESCs

In these new studies, we discovered that ESCs possess t-loops that are unaffected by the loss of TRF2. These TRF2-null, but looped, telomeres remain shielded from NHEJ but activate a mild ATM-dependent telomeric DDR. This DDR is attenuated both qualitatively and quantitatively relative to in TRF2-null somatic cells, as TRF2-null ESCs have dramatically fewer TIFs and do not undergo checkpoint arrest or cell death (Ruis et al. 2020). Indeed, unlike somatic cells in which the TRFH domain is required to inhibit ATM activation at telomeres, in ESCs apparently only the TRF2-iDDR domain is necessary to inhibit ATM activation (Ruis et al. 2020). Thus, as predicted by the t-loop model of end protection, t-loops that persist in the absence of TRF2 are sufficient to protect chromosome ends from NHEJ. This is the essential, long awaited evidence that conclusively demonstrates t-loops are a key mediator of end protection (de Lange 2018).

However, consistent with other reports that the repression of ATM and NHEJ are uncoupled, telomeres in TRF2-null ESCs still activate ATM, albeit weakly. The presence of t-loops is unable to completely protect chromosome ends from ATM activation in the absence of TRF2. This nicely complements recent findings showing that the persistent unwinding of t-loops activates ATM, despite the presence of TRF2 (Sarek et al. 2019). Together, these results demonstrate that t-loops are necessary but not sufficient for the complete inhibition of ATM at telomeres. Intriguingly, in somatic cells TRF2-iDDR has only previously been shown to repress NHEJ, downstream from ATM activation, while in ESCs TRF2-iDDR is necessary to suppress the activation of ATM itself (Okamoto et al. 2013; Van Ly et al. 2018). TRF2-iDDR interacts with MRN directly, providing one possible means through which TRF2-iDDR could impact upon ATM activation independently of t-loop formation.

Together, these new studies complement existing data to create a more complete picture of t-loop mediated end protection. The protection of chromosome ends requires t-loops, which both efficiently suppress NHEJ and significantly suppress ATM activation. The removal of t-loops leads to ATM activation, but if the other activities of TRF2 are retained, low levels of NHEJ. Likewise, the loss of these other functions of TRF2, most notably the TRF2 iDDR domain, can induce ATM activation but not NHEJ in the presence of t-loops. Thus, the presence of physiologically normal levels of t-loops is insufficient to completely suppress ATM in the absence of TRF2. Collectively therefore, protection from ATM and NHEJ is achieved via a two-step mechanism that involves t-loops and t-loop independent functions of TRF2. Loss of either t-loops or TRF2-mediated ATM inhibition alone leads to attenuated telomeric DDR signaling. However, the dual loss of t-loops and these t-loop independent functions of TRF2 unleashes complete telomeric DDR signaling involving robust ATM activation, NHEJ, cell cycle arrest and cell death (Fig. 5B,C).

In ESCs lacking TRF2, the mild telomeric ATM response is not accompanied by NHEJ, cell cycle arrest or dramatic loss of viability. This confirms previous reports that telomere fusions, cell death, and genomic instability do not accompany telomeric ATM activation *per se*, but rather the specific activation of NHEJ at telomeres. This is consistent with previous reports of an “intermediate state” of telomere end protection based on observations that in normal somatic cells the telomere DDR can be activated without accompanying telomere fusions (Cesare et al. 2009, 2013). In this context, the telomere DDR is thought to primarily serve as a signaling mechanism to identify shortened telomeres in aged somatic cells, arrest proliferation and trigger senescence (d'Adda di Fagagna et al. 2003; Kaul et al. 2012; Cesare et al. 2013). This has important implications as short telomeres in aged cells activate ATM but, apparently, very rarely activate NHEJ; telomere fusions in somatic tissues only arise upon complete erosion of the telomeric DNA substrate (d'Adda di Fagagna et al. 2003; Kaul et al. 2012; Cesare et al. 2013; Hayashi et al. 2015). Given t-loops require the exertion of topological stress onto ds telomeric DNA, presumably there is a minimum telomere length below which t-loops can no longer form. TRF2 would still be expected to suppress NHEJ, but not fully repress ATM, at short but linear telomeres, but critically short telomeres or telomere-free ends would lose TRF2 binding and protection completely. Could the activation of ATM but not NHEJ at short telomeres reflect this “intermediate state” of end protection and the loss of t-loops (Fig. 5D)?

## Outstanding questions regarding end protection in pluripotency

The observation that t-loop stabilization, protection from NHEJ and, largely, protection from ATM are achieved in a TRF2-independent manner in ESCs raises a number of key questions. Most prominently, how are t-loop formation and end protection achieved in pluripotent cells? Telomeres are highly repetitive and hence inherently recombinogenic, but since t-loop stabilization requires a specific mechanism in somatic cells, it seems unlikely that t-loops could be stable without similar assistance in pluripotent cells. The stabilization of t-loops by TRF2 does not involve ATP-hydrolysis or even telomere sequence specificity; the exposed lysines on the TRF2-TRFH domain interact with telomeric DNA in a sequence-independent manner and exert topological stress on the dsDNA, facilitating 3′ overhang invasion into this region (Amiard et al. 2007; Poulet et al. 2012; Benarroch-Popivker et al. 2016). T-loop formation therefore relies on the biochemical properties of TRF2 and the telomeric chromatin (Stansel et al. 2001). Since the same two major isoforms of TRF2 are expressed in ESCs and somatic cells, the TRF2 protein present at pluripotent and somatic telomeres is likely to possess the same biochemical properties, notwithstanding unknown post-translational modifications (PTMs). ESCs possess more open, less heterochromatic chromatin, which facilitates plasticity, maintaining ESC pluripotency while enabling the expression of lineage-specific genes during differentiation (Bernstein et al. 2006; Meshorer and Mistell 2006; Meshorer et al. 2006; Wen et al. 2009). These chromatin differences are observed at ESC telomeres, which possess reduced H3K9me^2/3^ and H4K20me^2/3^, and increased H3.3, relative to somatic cells (Marion et al. 2009; Wong et al. 2009, 2010). However, most components of telomeric chromatin, including the DNA sequence, shelterin, many shelterin cofactors, and chromatin modifiers, are apparently shared between ESCs and somatic cells. It seems unlikely, but not impossible, that these relatively minor differences would prevent TRF2 from stabilizing t-loops in ESCs. Regardless of whether TRF2 can or cannot stabilize t-loops in ESCs, since TRF2 is not required for this process other factor(s) must be involved. These factors could either act redundantly with TRF2, only becoming entirely responsible for t-loop stabilization when TRF2 is removed, or could be solely responsible for t-loop stabilization (if TRF2 is biochemically unable to mediate t-loop formation, due to differences in TRF2 PTMs or the telomeric chromatin discussed above). Analogously to TRF2 in somatic cells, these factors could promote t-loop stabilization by localizing to telomeres and exerting topological stress on telomeric DNA. Given this is a relatively nonspecific activity, a wide range of candidates could potentially perform this function. Alternatively, t-loops could be formed/stabilized in a different manner in ESCs, perhaps via an HR-like catalysis or direct D-loop stabilization mechanism, although this is entirely speculative and without precedent (Fig. 5C).

Clearly, finding the factors required for t-loop stabilization in ESCs is required to reveal the basis of the end protection mechanism. Several principles could guide this search. Factors involved in t-loop formation and end protection in ESCs should: be expressed in ESCs; localize to ESC telomeres; interact with DNA, directly or indirectly; be required for inhibition of telomeric ATM and NHEJ; be synthetic lethal with loss of TRF2, which would remove the t-loop-independent protective functions of TRF2. Given that the shelterin complex fulfils all these criteria, it is possible that another shelterin component could be directly or indirectly involved in t-loop formation in ESCs, perhaps by recruiting an unknown functional equivalent of TRF2. One specific alternative worthy of consideration is the telomeric repeat-containing RNA (TERRA), which is transcribed from and localizes to telomeres and is apparently up-regulated in ESCs (Azzalin et al. 2007). TERRA forms telomeric R-loops by displacing the G-rich strand and hence might apply topological stress to telomeres (Wang et al. 2015; Graf et al. 2017). Consistently, transcription of TERRA has been shown to promote t-loop formation in vitro (Kar et al. 2016). While this suggests transcription could have a role in t-loop formation in vivo, demonstrating such a link between TERRA and t-loop formation in vivo would be challenging and has yet to be achieved. Indeed, while the accepted model for t-loop formation in mammals involves a single invasion of the G-overhang to form a simple D-loop, other looped conformations could exist. For example *C.elegans* chromosomes end in t-loops despite their telomeres containing both 3′ and 5′ telomere extensions (Raices et al. 2008). One possibility is that both the 3′ and 5′ ends could invade the upstream telomeric sequences to form a more stable looped structure. This could explain how in vitro transcription of telomeric DNA leads to t-loops in the absence of overhangs, a situation that requires both ends of the telomeric DNA to be inserted into the upstream sequence (Kar et al. 2016; Tomáška et al. 2020).

Whatever the mechanism used for t-loop formation in ESCs, the questions of how and why this should specifically stabilize t-loops in pluripotent cells, and not in somatic cells, will also have to be addressed. If the factor(s) involved are amongst the many thousands of genes specifically expressed in the pluripotent state, addressing how this mechanism is restricted to pluripotent cells, but not necessarily why, will be relatively trivial (Young 2011). Currently, it seems this mechanism is restricted to the pluripotent stage of development; differentiation of TRF2-null ESCs induces telomeric NHEJ and ATM concomitant with their exit from pluripotency. However, understanding its components is necessary to confirm whether this mechanism of t-loop formation is relevant in other cell states; it should not be ignored that t-loops are still observed at 5%–10% of telomeres in TRF2-null somatic cells, albeit these “t-loops” could be linear telomeres misidentified as loops for technical reasons, as discussed above (Doksani et al. 2013; Van Ly et al. 2018). Could this mechanism be responsible for the partial stabilization of t-loops in TRF2-null somatic cells? Or could this mechanism be relevant in other stem cell states, including controversial cancer stem cells that apparently transcriptionally resemble ESCs and revert to an embryonic-like state (Ben-Porath et al. 2008; Chen et al. 2008; Chiou et al. 2008; Wang and Herlyn 2015)? Finally, it is worth noting that pluripotency is an acquired property; neither germ cells nor zygotes are pluripotent, but their descendants give rise to a subset of pluripotent cells in the developing embryo through a network of positive and negative feedback loops that gradually induce, then spatially restrict, pluripotency factor expression to the epiblast compartment. Thus, just as this largely TRF2-independent t-loop formation mechanism is apparently lost through differentiation, it must either be acquired in development or be present in the germ cells from which pluripotency ultimately derives. Given shelterin associates with germ cell-specific factors TERB1/MAJIN to facilitate attachment to the inner nuclear membrane during Meiosis, there is precedent for unique telomere transactions in the germ cell compartment and this should not be ignored (Daniel et al. 2014; Shibuya et al. 2015). However, with no evidence for TRF2-independent end protection in germ cells, it seems more parsimonious to suggest that this unique mode of telomere end protection might be linked to pluripotency factor expression and hence be acquired de novo in pluripotent cells, then rapidly lost from the pluripotent compartment upon lineage specification.

The depletion of TRF1 from ESCs induces telomeric replication stress phenotypes including ATR activation, reduced proliferation, telomere fragility and telomere loss, consistent with the crucial role of TRF1 in telomere replication described in somatic cells (Sfeir et al. 2009; Zimmermann et al. 2014; Porreca et al. 2020). Likewise, TPP1 or POT1 depletion from ESCs induces telomeric ATR activation, consistent with the described roles of TPP1 and POT1 in mediating G-overhang formation in somatic cells (Denchi and de Lange 2007; Kibe et al. 2010; Kibe et al. 2017). Therefore, with the exception of TRF2, shelterin components appear to have similar functions in somatic and pluripotent states. However, other aspects of telomere homeostasis evidently differ between these states. Most notably, removal of the t-loop unwinding helicase RTEL1 from somatic cells induces telomere loss and TC formation, as t-loops stall the replication fork and are then cleaved by SLX1/4 (Vannier et al. 2012, 2013). However, RTEL1-null ESCs show no evidence of either telomere loss or TC formation, suggesting t-loops in ESCs do not require RTEL1 for unwinding (Ding et al. 2004; Uringa et al. 2012). Is another helicase involved, or are these t-loops more flexible or dynamic, removing the need for an active t-loop unwinding mechanism in S phase? Likewise, TRF2 recruits APOLLO to initiate 3′ G-overhangs at leading end telomeres in somatic cells; failure of this axis leads to ATM activation and NHEJ at leading end telomeres (Wu et al. 2010; Wu et al. 2012). There is no evidence of NHEJ or shorter G-overhangs at leading end telomeres in TRF2-null ESCs. Do ESC telomeres employ a different mechanism to recruit APOLLO or a different exonuclease to initiate the 3′ G overhang at leading end telomeres? Evidently, aspects of telomere biology other than t-loop formation differ between pluripotent and somatic states and it would be worthwhile to consider the breadth of these differences.

## A dynamic model of t-loop-mediated end protection?

Except for newly replicated leading-end telomeres, which are blunt, all mammalian telomeres possess the fundamental features required for t-loop formation (double-stranded telomeric DNA and a terminal 3′ G overhang), so almost all telomeres could be looped. Moreover, t-loops are evidently an important component of telomere end protection. However, current studies only ever identify t-loops at ∼30% of telomeres, albeit this is likely an under-estimate for technical reasons. Given these observations, should we regard t-loops as the universal structure of mammalian telomeres, or could t-loops be just one structure occupied by telomeres? As discussed, mammalian telomeres must all enter a linear state at least once per cell cycle; t-loops are actively unwound by RTEL1 in S phase to enable passage of the replication fork, while DNA replication produces leading end telomeres with blunt ends that cannot form loops (Wu et al. 2010; Sarek et al. 2019). Access to the 3′ overhang is also required for telomerase to engage and extend telomere repeats, thus solving the end replication problem. Linear telomeres are obligatory during S phase and are therefore a relevant telomeric structure in vivo; despite the importance of the t-loop, not all telomeres form t-loops all the time. It is not known exactly how rapidly overhangs are generated on newly replicated leading end telomeres, but it is thought this process takes several hours (Chow et al. 2012). Likewise, it is unclear how quickly telomeres linearized by RTEL1 in S phase are restored to a looped structure. Regardless of exactly how long these telomeres are linear, they must remain protected from MRN and KU70/80, which bind very rapidly and efficiently to DNA ends and enact NHEJ on a time scale of seconds, prior to being restored to a looped conformation. This could explain the evolution of t-loop independent functions of TRF2, explicitly the roles of RAP1 and TRF2-iDDR in inhibiting the DDR at linear telomeres; linear telomeres exist in S phase and must remain protected from the DDR (Okamoto et al. 2013; Benarroch-Popivker et al. 2016).

However, linear telomeres could be more than a transient S-phase phenomenon. We recently demonstrated that a small proportion of telomeres in TRF2-null ESCs activate ATM (Ruis et al. 2020). Telomeric ATM activation typically requires both MRN and free DNA ends, suggesting these ATM-positive telomeres are linear (Lee and Paull 2005; 2007; Paull 2015; de Lange 2018). Since TRF2-null ESCs possess a similar frequency of t-loops to wild type ESCs and somatic cells, it therefore follows that some telomeres are linear in wild type somatic and pluripotent cells. When telomeres are forced to be linear for an extended period, for example by tethering of RTEL1 to telomeres by TRF2-S365A, ATM is activated and produces TIFs (Sarek et al. 2019). However, naturally occurring linear telomeres do not overtly activate ATM in normal cells, presumably as they exist only transiently and/or are protected by TRF2, as discussed above. One possibility is that the ATM-positive telomeres observed in TRF2-null ESCs exclusively represent telomeres transiently unwound in S phase. Alternatively, given that only 30%–35% of telomeres are ever observed to be in a t-loop conformation, it seems reckless not to consider the implication that linear telomeres might be a more abundant structure throughout the cell cycle. One possibility is that telomeres transiently pass through both looped and linear states as part of a dynamic equilibrium. This equilibrium would be controled by the competing activities of t-loop stabilizing factors such as TRF2, and factors that remove t-loops, including RTEL1. It could be that t-loops are the dominant state and each telomere transiently passes through a linear state once per cell cycle, during DNA replication. However, we propose that telomeres might dynamically transition between looped and linear states throughout normal cell cycling. Telomeres might pass through t-loop conformations sufficiently frequently to mask their ends from KU70/80 and MRN, while the t-loop independent functions of TRF2, TRF2-iDDR-mediated inhibition of NHEJ and/or ATM and RAP1-mediated inhibition of NHEJ, maintain protection in the linear state. While linear telomeres can be transiently protected from ATM and NHEJ, prolonged telomere linearity, for example, in cells expressing TRF2-S365A or at short telomeres produced through aging, causes the loss of efficient repression of ATM, suggesting telomeres must pass through a looped state for continued inhibition of ATM. This presumably explains why active t-loop unwinding by RTEL1 is so tightly controled in S phase: Promiscuous t-loop unwinding would lead to unwarranted ATM activation. The frequency of t-loops might vary between cells in a population, perhaps in a cell cycle-dependent manner, or indeed individual telomeres might form t-loops with different frequencies, depending perhaps on their three-dimensional position within the nucleus, or the sequence of their subtelomeric region. New techniques to visualize t-loops within individual, ideally live, cells, rather than looking at t-loops in fixed DNA obtained from a large pool of cells, is required to test this dynamic model and address the true frequency of t-loop formation in vivo (Fig. 5B,C).

## Conclusions

While experiments that produce a static picture are undoubtedly useful for understanding the basic components and functionalities of a biological system, it is clear that biological systems are highly dynamic, typically contain significant redundancy and often involve cooperation between multiple components. By way of analogy, imagine a football/soccer match. One could learn many aspects of the game from a single photographic snapshot (22 players, one ball, a referee, goal posts, the pitch, etc.), but to understand the rules by which the game is played (passing between players, the referee, the offside rule, etc.) one would need video footage. Likewise, experiments on pooled, fixed material provide useful information regarding the components of a system but cannot reflect its complexity. Telomeres are no exception; classical experiments have revealed many of the components required for end protection and how these individually restrict DDR activities. For example, we know that t-loops, TRF2, the TRF2-iDDR domain, RAP1, Apollo, MRN, and KU70/80 all cooperate to protect chromosome ends from ATM and NHEJ. However, we still have little idea of how exactly these activities are coordinated in vivo. Are t-loops truly dynamic? Do specific chromosome ends show preferences for t-loop or linear states? Do these states vary throughout the cell cycle? The development of new telomere-specific approaches and co-opting assays from related fields is now required to produce a more complete, three-dimensional understanding of how telomeres solve end protection.

While it is now clear that aspects of end protection differ between pluripotent and somatic states, why this should be remains unknown. If TRF2-mediated t-loop formation is necessary to protect somatic chromosome ends, why is this not the case in pluripotent cells? Has an alternative mechanism evolved to specifically protect chromosome ends in early development? Is TRF2-independent t-loop stabilization a mere accident, only revealed by the experimental removal of TRF2, something that would never occur in development and hence never be observed by evolution? Identifying the mechanisms of t-loop stabilization and end protection in pluripotent cells should hopefully help elucidate this teleological question.
